# Calibrated cardiac output monitoring versus standard care for fluid management in the shocked ICU patient: a pilot randomised controlled trial

**DOI:** 10.1186/s40560-018-0356-y

**Published:** 2019-01-10

**Authors:** Timothy G. Scully, Robert Grealy, Anthony S. McLean, Sam R. Orde

**Affiliations:** Nepean Hospital, Kingswood, NSW Australia

**Keywords:** Fluid responsiveness, Cardiac output monitoring, Minimally invasive, Shock, Sepsis and fluid administration

## Abstract

**Background:**

Despite the evidence for calibrated cardiac monitored devices to determine fluid responsiveness, there is minimal evidence that the use of cardiac output monitor devices leads to an overall change in IV fluid use. We sought to investigate the feasibility of performing a randomised controlled study using calibrated cardiac output monitoring devices in shocked ICU patients and whether the use of these devices led to a difference in total volume of IV fluid administered.

**Methods:**

We performed a single-centre non-blinded randomised controlled study which included patients who met the clinical criteria for shock on admission to ICU. Patients were divided into two groups (cardiac output monitors or standard) by block randomisation. Patients allocated to the cardiac output monitor all received EV1000 with Volume View sets. Daily intravenous fluid administration and cumulative fluid balance was recorded for 3 days. The primary outcome assessed was the difference in daily intravenous fluid administration and cumulative fluid balance at 72 h between the two groups. We also assessed how often the clinicians used the cardiac monitor to guide fluid therapy and the different reasoning for initiating further intravenous fluids.

**Results:**

Eighty patients were randomised and 37 received calibrated cardiac output monitors. We found no adverse outcomes in the use of calibrated cardiac output monitoring devices and that was feasible to perform a randomised controlled trial. There was no significant difference between the standard care group vs the cardiac monitoring group for cumulative fluid balance (2503 ± 3764 ml vs 2458 ± 3560 ml, *p* = 0.96). There was no significant difference between the groups for daily intravenous fluid administration on days 1, 2 or 3. In the cardiac monitored group, only 43% of the time was the EV1000 output incorporated into the decision to give further intravenous fluids.

**Conclusion:**

It is feasible to perform a randomised controlled trial using calibrated cardiac output monitoring devices. In addition, there was no trend to suggest that the use of a cardiac monitors leads to lower IV fluid use in the shocked patient. Further trials will require study designs to optimise the use of a cardiac output monitor to determine the utility of these devices in the shocked patient.

**Trial registration:**

ANZCTR, ACTRN12618001373268. Registered 15 August 2018—retrospectively registered.

**Electronic supplementary material:**

The online version of this article (10.1186/s40560-018-0356-y) contains supplementary material, which is available to authorized users.

## Background

A pillar of critical care medicine that has come under examination recently is the use of intravenous (IV) fluids for volume resuscitation in critically ill patients. Over the past 18 years, multiple studies have illustrated the association between positive fluid balance and morbidity and mortality[1–5]. One prominent feature of these trials is that a prolonged fluid balance at the end of day 1 to 4 following the onset of critical illness leads to the increase in mortality; not necessarily the volume given in the initial resuscitation period [3, 4, 6, 7]. In contrast, some studies have shown that critically ill patient who are ‘unfilled’ have worse outcomes [8].

The challenge of determining the optimum fluid volume to be administered to critically ill patients is well known. Usually such patients receive liberal administration of fluids following presentation to hospital yet accurately evaluating ongoing fluid requirements can be difficult in the face of ongoing hypotension and oliguria. Due to the complex haemodynamic changes in the state of shock, judging fluid balance by conventional means is highly challenging [4, 9].

As illustrated by the FENICE trial in 2015, despite evidence to the contrary, most clinicians will still base their decision to give fluids on the presence of hypotension or static parameters, including single measurement central venous pressures (CVP)[10]. The use of either hypotension or CVP to judge fluid status/responsiveness has been shown to be barely better than a coin flip [3, 11, 12].

Several dynamic modalities have been used to assess fluid responsiveness in the critically ill patient. The use of devices employing transpulmonary thermodilution to assess fluid responsiveness such as cardiac output/index (CO/CI), stroke volume variation (SVV) and other derived haemodynamic parameters have gained in popularity [13–16]. The advantage of these devices is intermittent calibration by thermodilution combined with pulse contour analysis which has been suggested to be more accurate in the critically ill population than non-calibrated devices in predicting fluid responsiveness [17].

Despite the evidence for calibrated cardiac monitored devices to determine fluid responsiveness, there is minimal support that the use of cardiac output monitor devices leads to an overall change in IV fluid use. Furthermore, it is still to be determined if the ability to more accurately predict fluid responsiveness correlates with improved fluid administration.

We sought to perform a pilot study to assess (i) feasibility of performing a randomised controlled study in our unit using calibrated cardiac output monitoring devices, (ii) whether the use of these devices led to a difference in volume of IV fluid used and (iii) if there is a difference in clinician rationale for giving fluid boluses when a cardiac output monitor device was present.

## Methods

We conducted a non-blinded, randomised controlled study at the Nepean Hospital Intensive Care Unit (ICU), Sydney, NSW, Australia from December 2016 to January 2018. The study was approved by the Nepean Blue Mountains Local Health District Research Governance Office (Study 16/54–HREC/16/NEPEAN/89). Consent for participation was waived as cardiac output monitoring does not require additional invasive procedures to establish, and its use is part of standard therapy in our unit.

Inclusion criteria were adult patients (18 years or older) admitted to the ICU with, or developed, shock defined as ongoing hypotension, despite fluid resuscitation, requiring vasopressors to maintain mean arterial blood pressure (MAP) ≥ 65 mmHg + and a serum lactate ≥ 2 mmol/l.

Exclusion criteria were patients in hypovolemic/haemorrhagic shock or atrial fibrillation (AF), pre-existing need for dialysis, pregnancy and patients who were unlikely to remain in the ICU for at least 72 h. Additionally, patients were excluded if advanced haemodynamic monitoring was considered essential by the treating clinician.

Those randomised to the cardiac output monitoring group received an EV1000 in combination with Volume View (Edwards Lifescience, USA). Volume View allows for continuous and intermittently calibrated measurements of CO, CI and SVV. The calibration is performed by transpulmonary thermodilution, requiring a subclavian or internal jugular central line and a femoral arterial line equipped with a thermistor. While the EV1000 can be setup to utilise both inflatable finger cuff monitoring (Nexfin) and non-calibrated arterial waveform analysis (FloTrac), both methods have been shown to be unreliable in the critically ill (11). Therefore, Volume View was mandatory for all patients randomised to the cardiac output monitor group.

### Study design

Patients identified for inclusion were randomised within the first 24 h of development of shock to either the standard care group or to the cardiac output monitoring group. Block randomisation was performed via an independent statistician with block size of 4 and 1:1 allocation. Patients allocated to the cardiac output monitor all received EV1000 with Volume View sets. For patients allocated to standard monitoring, the treating ICU team could use any method of assessing fluid status/responsiveness commonly utilised in ICU. In the cardiac output monitoring group, despite the presence of an EV1000, the treating team were not mandated to utilise the EV1000 parameters and were free to use non-EV1000 data to guide fluid use. An independent investigator (TS) recorded total IV fluids given and total fluid balance at 24, 48 and 72 h. Clinicians who decided a fluid bolus was necessary during the 72-h study period were asked to record the following: reason, volume and type of fluid given, and also what parameters the clinician used to judge the success of the fluids given (see Additional file 1 for example of questionnaire provided).

### Data and statistical analysis

Patient data collected included demographic and physiological data, past medical history of including arrhythmias, congestive heart failure and renal failure. Fluid input and output was documented via the ICU electronic data entry system. Daily fluid balances were calculated using fluid input and output measurements. Daily IV fluids were defined as the IV fluids used in 24 h for fluid replacement and excluded other forms of fluid input (i.e. nutritional or drug delivery). The daily IV fluid use was calculated to ensure any observed difference in fluid balance between the two groups was due to fluids used as a therapy and not a confounder from higher rates of other IV fluids.

Sample size estimation using data from our unit by Pittard et al. (2017) with an effect size of 300 ml and standard deviation of 2000 ml, determined that 697 patients would be required for a full study [1]. Given this was a pilot trial to determine feasibility, we aimed to recruit at least 10% of this calculated number, as per pilot sample size guidelines established by Cocks and Torgerson (2013, [18]).

Statistical analysis was performed with SPSS version 24 (IBM Corp, Armonk, New York). Continuous variables are reported as mean ± standard deviation (SD) or median ± interquartile range (IQR) if not normally distributed. Categorical variables are expressed as number of patients and percentage of group. Comparisons for categorical data was made by Pearson’s chi-squared test or Fisher’s exact test if less than five patients were in a specific group. For continuous variables, independent *t* test was used for normally distributed data and a Mann-Whitney *U* test for non-parametric data. Probability values are considered two-sided and a *p* value < 0.05 was considered significant. Statistical analysis was performed on an intention to treat basis.

## Results

### Demographics

**Fig. 1:**
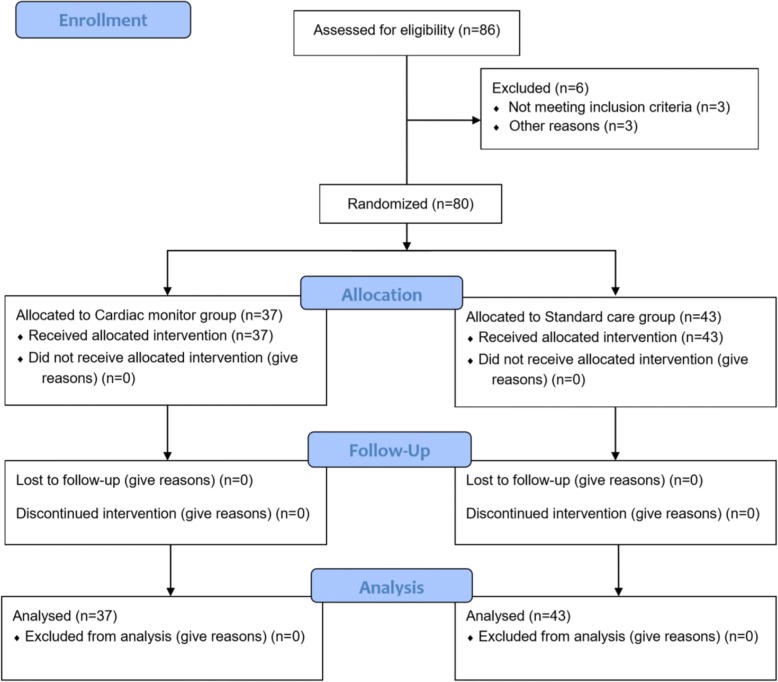
Flow chart of subject enrolment

From December 2016 to January 2018, 86 patients were recruited into this study. The flow chart for enrolment is shown in Fig. 1. A total of 80 patients were included in the final analysis.

The median age of patients in the study was 64 years old and 49% of the cohort was male. The majority of shock was due to sepsis (73%) and cardiogenic (17%), with the remaining 10% due to other causes. Twenty-three patients died while in the ICU (29% mortality). Median length of stay was 9 days (IQR 1 to 17 days). There was no significant difference between the standard and cardiac monitored group in any baseline parameter. There was also no significant difference in MAP or lactate at 24, 48 or 72 h between the two groups (Table 1*)*.

**Table 1:** Demographic characteristics, morbidity and mortality data of cardiac monitor and standard monitored patients

Variable	All patients (*n* = 80)	Standard (43)	Cardiac monitor (37)	*p* value
Demographics				
Age (years)*	64.41 (56–72)	65.81 (58–72)	62.78 (52–72)	0.437
Sex (male, *n*)	49 (61)	26 (61)	23 (62)	0.880
Weight (kg)*	88.00 (75–101)	85.14 (74–96)	91.23 (75–107)	0.478
Past medical history				
Chronic renal failure (*n*)	7 (9)	4 (9)	3 (8)1	1.000
Congestive heart failure (*n*)	4 (5)	3 (7)	1 (3)	0.630
Type of shock				
Septic shock (*n*)	58 (73)	32 (74)	26 (70)	0.618
Cardiogenic shock (*n*)	14 (17)	6 (43)	8 (57)	0.368
Other causes of shock/undetermined (*n*)	8 (10)	5 (12)	3 (8)	0.601
Biochemistry/haemodynamics				
MAP at time of enrolment (mmHg)	64.54 ± 6.2	64.81 ± 5.4	64.21 ± 7.1	0.671
Bicarbonate (mmol/l)	18.63 ± 5.7	19.6 ± 4.8	18.3 ± 5.4	0.273
Base excess (mEq/l)	− 6.89 ± 6.0	− 5.8 ± 5.9	− 7.43 ± 5.9	0.228
Bilirubin (mol/l)*	13 (8–18)	12 (5–19)	12 (1–23)	0.702
Haemoglobin (g/l)	111.95 ± 20.9	113.3 ± 25.1	114.1 ± 26.3	0.885
Platelets (×10^9^/l)	214.53 ± 155.5	184.0 ± 101.3	209.2 ± 133.4	0.340
Blood sugar level (mmol/l)	9.26 ± 4.6	8.3 ± 4	9.5 ± 5.2	0.268
Creatinine (mol/l)*	130 (55–205)	125 (55–195)	143 (75–211)	0.969
Morbidity/mortality				
24-h MAP (mmHg)	72.10 ± 11.0	73.00 ± 9.8	71.25 ± 12.2	0.492
48-h MAP (mmHg)	76.73 ± 15.0	74.64 ± 14.8	78.73 ± 15.1	0.355
72-h MAP (mmHg)	75.29 ± 10.9	71.15 ± 8.8	79.14 ± 11.6	0.056
Day 1 lactate (mmol/l)*	2.35	2.20	2.60	0.896
Day 2 lactate (mmol/l)*	1.50	1.50	1.60	0.456
Day 3 lactate (mmol/l)*	1.30	1.30	1.20	0.346
Ventilated (*n*)	51 (64%)	26 (70%)	25 (58%)	0.260
ICU stay (days)*	9.71 (5–13)	9.40 (6–12)	10.08 (6–14)	0.799
Patients requiring dialysis (*n*)	17 (21)	6 (14)	11 (30)	0.085
Death (*n*)	23 (29)	11 (26)	12 (32)	0.622

Results for normal distributed data given as mean ± standard deviation or frequency (percentage). *Non-normally distributed data presented as median (25th centile, 75th centile)

### Total intravenous fluids and fluid balance

**Fig. 2:**
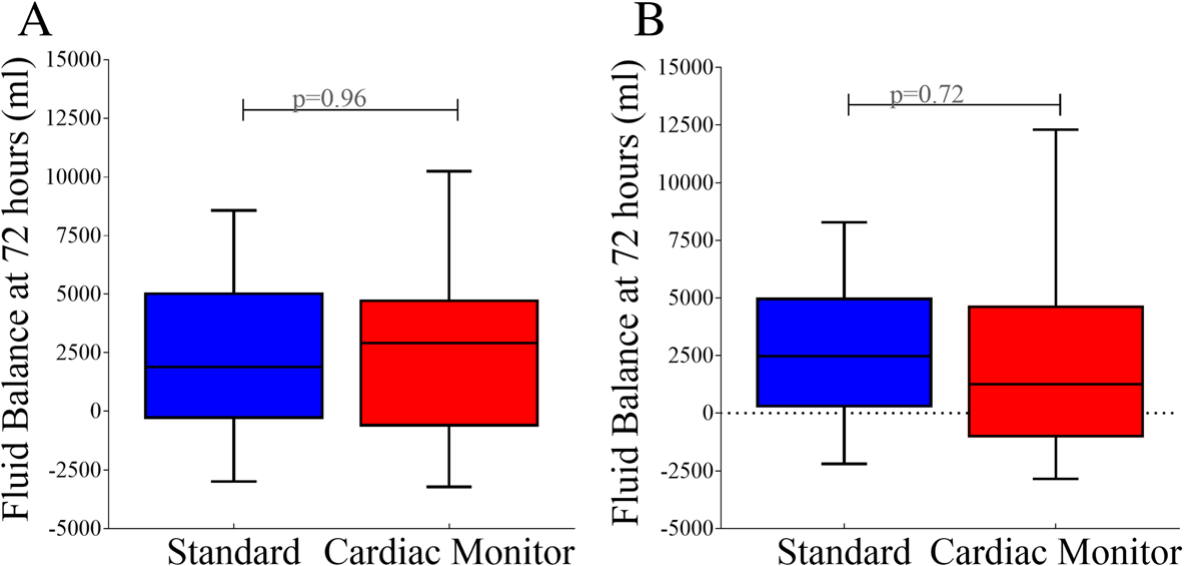
Fluid balance at 72 h. Legends: box plot comparing the average fluid balance at 72 h following randomisation between cardiac output monitored patients and standard monitored patients in **a** all patients and **b** septic shock patients

When comparing fluid balance at time of discharge, death or at 72 h (whichever came first), there was no significant difference between the standard care group vs the cardiac monitoring group (Fig. 2a). The average fluid balance of the standard group was 2503 ± 3764 ml, and the cardiac monitoring group had an average fluid balance of 2458 ± 3560 ml. As most of data regarding excess fluids comes from the septic population, in addition analysis was performed on septic patients alone (Fig. 2b). In this septic shock population, the standard group had an average fluid balance of 2642 ± 543 ml while the cardiac output monitoring group had an average fluid balance of 2311 ± 763 ml at 72 h (*p* = 0.716).

**Fig. 3:**
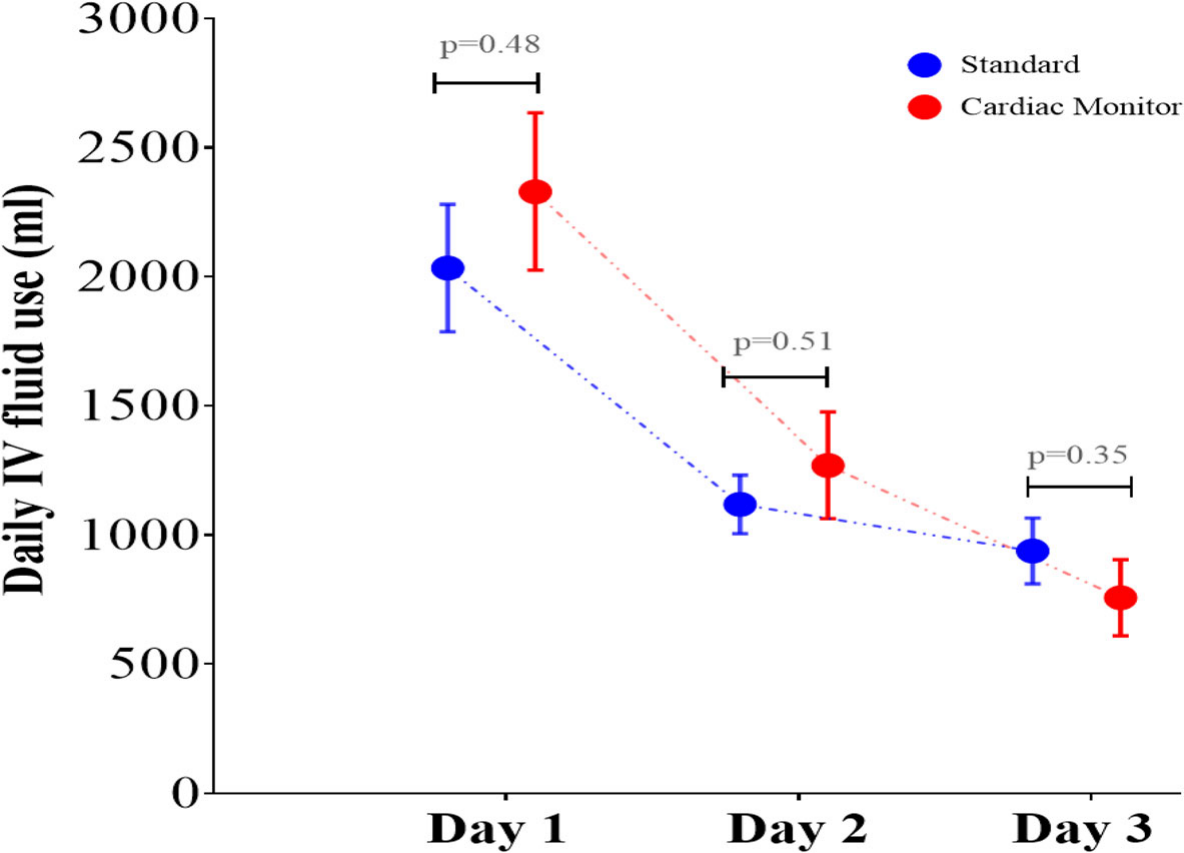
Daily IV fluid use on days 1, 2 and 3. Comparison of days 1, 2 and 3 average total IV fluid use between cardiac output monitored and standard monitored group and demonstrating trend in total daily IV fluid use from day 1 to day 3 for each group

Figure 3 demonstrates the total volume of IV fluids given on days 1, 2 and 3. As illustrated in Fig. 3, there was no significance for daily IV fluid volumes between the two groups. On day 1, the cardiac monitoring group received more IV fluids (2330 ± 304 ml vs 2034 ± 247 ml). On day 2, the cardiac monitoring group received on average 1270 ± 206 ml of IV fluids compared to the standard group 1119 ± 114 ml. On day 3, the standard care group received more IV fluid (938 ± 128 ml vs 757 ± 148 ml).

Although 37 patients were randomised to the cardiac output monitoring group and equipped with an EV1000, the parameters derived from the EV1000 was only incorporated into the decision-making for further IV fluids for 27 of these patients. When analysis was performed based on comparison between patients where the EV1000 parameters were utilised and all other patients, there was a significantly higher MAP at 72 h for the EV1000-guided patients. However, there remained no significant change in fluid balance at 72 h, mortality, ICU length of stay, ventilation or dialysis requirement (see Additional file 2).

### Reason for fluid boluses and assessment of fluid bolus success

Regarding the secondary aim of this study, Table 2 characterises the use of fluid boluses in each of the two groups. Over 72 h, the standard monitoring group had on average one fluid bolus while the cardiac monitoring group received a mean of two fluid boluses. Nearly all boluses administered during the study were either 250 or 500 ml of either a crystalloid or albumin 4%.

**Table 2:** Comparison of assessment for fluid boluses and fluid responsiveness between cardiac and standard monitored patients

Variable	Total	Standard	Cardiac monitor	*p* value
Fluid boluses				
Average number of boluses (*n*)*	1 (0.4)	1 (0.4)	2 (0.5)	0.237
Total fluid given as boluses (ml)*	500 (0–1200)	500 (0–1250)	500 (0–1130)	0.234
Reason for giving fluid bolus^#^				
Number of fluid boluses	114			
Hypotension (*n*)	52 (46)	32 (58)	20 (35)	*0.015*
Urine output (*n*)	15 (13)	11 (20)	4 (7)	0.055
Tachycardia (*n*)	8 (7)	5 (9)	3 (5)	0.486
Lactate (*n*)	15 (13)	14 (25)	1 (2)	*0.001*
Clinical exam findings (*n*)	2 (2)	2 (4)	0	0.239
Increasing noradrenaline requirement	36 (31)	12 (21)	24 (41)	*0.022*
TTE assessment	16 (14)	10 (18)	6 (10)	0.248
SVV, CI, CO, EVLW or GEDV	25 (22)	0	25 (43)	*0.001*
Passive leg raise	4 (4)	3 (5)	1 (2)	0.360
Assessment of response to fluid				
No assessment of response to fluid (*n*)	49 (43)	25 (45)	24 (41)	0.725
Urine output (*n*)	6 (5)	2 (4)	4 (7)	0.427
Change in MAP (*n*)	24 (21)	19 (34)	5 (9)	*0.001*
Change in NR requirements (*n*)	32 (28)	11 (20)	21 (36)	*0.049*
SVV, CI, CO, EVLW or GEDV	15 (13)	0	15 (26)	*0.001*

Results for normal distributed data given as frequency (percentage)

^*^Non-normally distributed data presented as median (25th centile, 75th centile). *TTE* transthoracic echocardiogram, *SVV* stroke volume variation, *CI* cardiac index, *CO* cardiac output, *EVLW* extra-vascular lung water, *GEDV* global end-diastolic volume

^*#*^Clinicians may have recorded multiple reasons for each bolus, each reason has been scored. Italicised *p* values indicated significant difference between the two groups

The significant differences between the two groups were the standard monitoring group had a significantly higher percentage of fluid boluses given for hypotension (58% vs 35%, *p* = 0.015) and a trend in serum lactate (25% vs 2%, *p* < 0.001). In contrast, the cardiac output monitoring group clinicians utilised the EV1000 parameters ((43% vs 0%, *p* < 0.0001) and increasing vasopressor requirements (41% vs 21%, *p* = 0.022) as a guide for further fluid boluses more frequently than the standard monitoring group. The justification of whether a fluid bolus had been successful was similar to the initial reason to give fluids within each group. Change in blood pressure was used for 34% of the standard monitoring group and only 9% of the cardiac output monitoring group (*p* < 0.001). The response to fluid bolus in the cardiac output monitoring group was judged by change in EV1000 parameters and change in vasopressor requirements at a significantly higher rate than in the standard monitoring group. However, for 43% of fluid boluses over both groups, there was no recorded assessment of a parameter of interest following fluid bolus administration.

## Discussion

We found that a randomised controlled study using cardiac output monitoring devices was feasible. We demonstrated that in the 80 patients included, there was no association between the use of minimally invasive cardiac output monitoring devices and total volume of IV fluids administered or cumulative fluid balance over a 72-h period. The same trend was seen when only septic shock patients were analysed. There was no significant difference between standard monitoring and patients with a cardiac output monitor for IV fluid volumes received on days 1, 2 or 3.

When reviewing the reasons behind why doctors chose to give fluid boluses, a number of interesting trends emerge. The presence of hypotension was used in 46% of all cases as one of the primary rationale to justify that further fluids were required. The other most commonly cited rationale, used in 31% of all cases for a fluid bolus in either group, was an increasing vasopressor requirement. In the cardiac output monitoring group, the use of the cardiac monitor in 43% of cases was only slightly more commonly used than an increase in noradrenaline requirements (41%) and hypotension (35%). Over one third of cardiac monitoring cases still focused on hypotension as a reason to give fluid when a cardiac monitor was present. A similar picture is seen when looking at how doctors judged the success of their fluid bolus, with hypotension and change in vasopressor requirement the leading reasons between the two groups.

This underlines that clinicians continue to target blood pressure as an endpoint for fluid boluses despite the evidence that fluid bolus therapy with an aim to resolve hypotension has limited use [19]. Using increasing vasopressor requirements as justification for fluid administration is problematic for two reasons. Firstly, an increasing vasopressor requirements may not indicate hypovolemia but a worsening clinical state due to multiple factors and should not necessarily be followed by a fluid bolus. In addition, vasopressors are usually started once adequate fluid resuscitation has been achieved, making it less likely a patient will respond to further IV fluids.

There are several possible explanations for why there was no difference between the two groups with regard to fluid balance. The most likely explanation is a lack of physician interest and comprehension of dynamic parameters of fluid responsiveness, resulting in non-compliance. This is well illustrated in Table 2, where despite the presence of an EV1000 in the cardiac output monitoring group, dynamic parameters were incorporated in less than half of the decisions to give a fluid bolus. This information agrees with data presented in the FENICE study, which showed that hypotension and static markers of fluid responsiveness were used far more often than dynamic markers as reasons to give fluid boluses [10]. One interesting additional finding our study demonstrates is that even when dynamic indices are readily available, ICU doctors continue to utilise parameters such as urine output or blood pressure to judge fluid status/responsiveness. This is not an individual issue in our unit; Rameau (2017) performed a similar study to analyse if passive leg raising (PLR) in conjunction with calibrated cardiac monitoring resulted in a lower 48 h fluid balance [15]. Using an increase in stroke volume of 10% following PLR, they determined 75% of their patients were not fluid responsive. However, 54% of these fluid non-responsive patients received IV fluids. After re-education of their staff and repeat analysis, a significant decrease in fluid balance was seen [15]. As previously suggested, the reasons behind non-compliance are multi-factorial and likely centres around the heuristic technique for problem-solving [10, 15, 20].

Higher cumulative fluid balances have been reported in other studies. Sadaka (2014) reported a day 1 fluid balance of 6.5 l and Boyd (2011) had average cumulative 96-h fluid balance of 11 l [2, 3]. This may be due to the trend of lower fluid use in our ICU. In a paper by Pittard (2017), analysing the association of fluid balance and mortality in sepsis and septic shock, which was conducted in the same ICU as this trial, the daily fluid balance on day 1 was 1.4 l [1]. A larger sample size will be required to detect a clinically significant difference if lower overall volumes are used.

Finding no observed difference in total IV fluid use or overall fluid balance when a cardiac output monitoring device was used may be due to the underlying principle of how we define fluid responsiveness being flawed. As shown by multiple studies, while patients who are deemed to be fluid responsive have a significant increase in cardiac output at 1-min post IV fluid challenge, over time this effect will dissipate and after 90 min, there will be no difference between fluid responders and non-responders [21, 22]. This effect may be compounded in septic patients, with even less of the fluid bolus remaining in the intravascular space [23]. It may be that relying on macro-haemodynamic monitoring in the critically ill will remain an unreliable method of judging fluid administration and further investigation into microvasculature monitoring is required.

Strengths of our study include a large sample size for a pilot study and the randomisation process. In addition, the ratio of septic shock to cardiogenic and other forms of shock in this trial is in agreement with other studies, which have found similar proportions of causes of shock [24]. This should increase the external validity of this trial. However, the use of multiple causes of shock in a small single-centre pilot trial may also be a weakness as the different haemodynamic effects of different forms of shock leads to a wide range of total IV fluids used.

Future studies should take note of the non-compliance issue regarding fluid responsiveness assessment, and study designs should be designed to minimalize this effect. Ultimately, how we monitor fluid balance on the macro haemodynamic level may not change the amount of fluid given to the shocked patient. Instead, re-education is needed for how fluids are viewed, from a harmless intervention to one that has risk if too much or too little is provided. A paradigm shift into how the shocked patient is managed is required to solve the issue of how best to determine the optimal volume of IV therapy required.

## Conclusion

In this pilot study, we found that it was feasible to perform a randomised controlled trial using these devices. In addition, there was no trend to suggest that the use of an EV1000 and Volume View device leads to lower IV fluid use in the shocked patient. A follow up trial with a design to optimise the use of a cardiac output monitor is necessary to determine the utility of these devices in the critically ill.

## Additional files

Additional file 1: Example of data sheet completed by clinicians to assess reasons for fluid boluses. (PDF 116 kb)

Additional file 2: Comparison of fluid balance and clinical outcomes between patients where haemodynamic monitoring was actively used and all other patients where haemodynamic montioring was not actively used. (DOCX 18 kb)

## Abbreviations

AF: Atrial fibrillation
CI: Cardiac index
CO: Cardiac output
CVP: Central venous pressures
ICU: Intensive care unit
IQR: Interquartile range
IV: Intravenous
MAP: Mean arterial blood pressure
PLR: Passive leg raising
SD: Standard deviation
SVV: Stroke volume variation
