# Supplementary material for: Calibrated cardiac output monitoring versus standard care for fluid management in the shocked ICU patient: a pilot randomised controlled trial
Source: J Intensive Care. 2019 Jan 10;7:1. doi: 10.1186/s40560-018-0356-y (PMC6329049; doi:10.1186/s40560-018-0356-y)
Supplement: Supplementary file 1 — Example of data sheet completed by clinicians to assess reasons for fluid boluses. (PDF 116 kb) [file 40560_2018_356_MOESM1_ESM.pdf]

CHAMS Study - Clinical data collection sheet

**Patient**	ID CODE	
	Date of admission	/ /
	Time of admission	/ /

Fluid bolus record

Bolus 1

Clinician (circle)	JMO/Registrar/SR/Fellow/Specialist		
Date	/ /		
Time	:		
Clinical trigger	Clinical parameter	☐ Hypotension	mm Hg
	(select relevant parameter and	☐ Tachycardia	bpm
	indicate value)	☐ Oliguria	mls
		☐ JVP/CVP	
		☐ Lactate	mmol/L
		☐ Echo (specify parameter)	
		☐ Other clinical parameter (detail)	
	Fluid responsivenes parameter	☐ Passive leg raise	
	(select relevant parameter and	☐ Stroke vol variation	%
	indicate value)	☐ Pulse pressure variation	%
Fluid	Fluid type		
	Volume	mls	
	Duration over	mins	
	Response		

Bolus 2

Clinician (circle)	JMO/Registrar/SR/Fellow/Specialist		
Date	/ /	Time	:
Time	:		
Trigger	Clinical parameter	☐ Hypotension	mm Hg
	(select relevant parameter and	☐ Tachycardia	bpm
	indicate value)	☐ Oliguria	mls
		☐ JVP/CVP	
		☐ Lactate	mmol/L
		☐ Echo (specify parameter)	
		☐ Other clinical parameter (detail)	
	Fluid responsivenes parameter	☐ Passive leg raise	
	(select relevant parameter and	☐ Stroke vol variation	%
	indicate value)	☐ Pulse pressure variation	%
Fluid	Fluid type		
	Volume	mls	
	Duration over	mins	
	Response		

Bolus 3

Clinician (circle)	JMO/Registrar/SR/Fellow/Specialist		
Date	/ /		
Time	:		
Trigger	Clinical parameter	☐ Hypotension	mm Hg
	(select relevant parameter and	☐ Tachycardia	bpm
	indicate value)	☐ Oliguria	mls
		☐ JVP/CVP	
		☐ Lactate	mmol/L
		☐ Echo (specify parameter)	
		☐ Other clinical parameter (detail)	
	Fluid responsivenes parameter	☐ Passive leg raise	
	(select relevant parameter and	☐ Stroke vol variation	%
	indicate value)	☐ Pulse pressure variation	%
Fluid	Fluid type		
	Volume	mls	
	Duration over	mins	
	Response		
