# Supplementary material for: Calibrated cardiac output monitoring versus standard care for fluid management in the shocked ICU patient: a pilot randomised controlled trial
Source: J Intensive Care. 2019 Jan 10;7:1. doi: 10.1186/s40560-018-0356-y (PMC6329049; doi:10.1186/s40560-018-0356-y)
Supplement: Supplementary file 2 — Comparison of fluid balance and clinical outcomes between patients where haemodynamic monitoring was actively used and all other patients where haemodynamic montioring was not actively used. (DOCX 18 kb) [file 40560_2018_356_MOESM2_ESM.docx]

| **Variable** | **Patients without active cardiac monitoring (n=53) #** | **Patients with active use of cardiac monitoring (n= 27)** | **P value** |
| --- | --- | --- | --- |
| Fluid balance day 3 (mL) | 2507 ± 3514 | 2434 ± 4009 | 0.934 |
| 24-hour MAP (mmHg) | 71.15 ± 11.8 | 74.09 ±8.8 | 0.305 |
| 48-hour MAP (mmHg) | 75.06 ± 14.5 | 79.52 ± 8.8 | 0.309 |
| 72-hour MAP (mmHg) | 71.88 ± 8.7 | 81.10 ± 12.2 | **0.031** |
| Day 1 Lactate (mmol/L) * | 2.35 | 2.30 | 0.231 |
| Day 2 Lactate (mmol/L) * | 1.40 | 1.65 | 0.695 |
| Day 3 Lactate (mmol/L) * | 1.30 | 1.25 | 0.140 |
| ICU stay (days) * | 5.5 (1.5-9.5) | 7 (4-10) | 0.879 |
| Death (n) | 16 (30) | 7 (27) | 0.764 |

**Legend:** Results for normal distributed data given as mean ± standard deviation or frequency (percentage). *Non-normally distributed data presented as median (25^th^ centile, 75^th^ centile).

# Of the 53 patients, 43 are of the standard group randomized to not receive a cardiac monitoring device and 14 are patients randomized to the cardiac monitoring group who had no evidence that the cardiac monitor was used to guide fluid management decisions.
